# Olodaterol exerts anti-inflammatory effects on COPD airway epithelial cells

**DOI:** 10.1186/s12931-021-01659-2

**Published:** 2021-02-23

**Authors:** Nan Yang, Gurpreet K. Singhera, Yi Xuan Yan, Michael P. Pieper, Janice M. Leung, Don D. Sin, Delbert R. Dorscheid

**Affiliations:** 1 Center for Heart Lung Innovation, St. Paul’s Hospital, University of British Columbia, Vancouver, BC Canada; 2 Boehringer Ingelheim Pharma GmbH & Co. KG, Ingelheim am Rhein, Germany

**Keywords:** Airway epithelium, Interleukin-8, Airway inflammation, Olodaterol, Long-acting beta agonist, COPD, Beta 2-adrenergic receptor

## Abstract

**Background:**

Airway inflammation is a key feature of chronic obstructive pulmonary disease (COPD) and inhaled corticosteroids (ICS) remain the main treatment for airway inflammation. Studies have noted the increased efficacy of ICS and long-acting beta 2 agonist (LABA) combination therapy in controlling exacerbations and improving airway inflammation than either monotherapy. Further studies have suggested that LABAs may have inherent anti-inflammatory potential, but this has not been well-studied.

**Objective:**

We hypothesize that the LABA olodaterol can inhibit airway inflammation resulting from exposure to respiratory syncytial virus (RSV) via its binding receptor, the β2-adrenergic receptor.

**Methods:**

Human bronchial epithelial brushing from patients with and without COPD were cultured into air–liquid interface (ALI) cultures and treated with or without olodaterol and RSV infection to examine the effect on markers of inflammation including interleukin-8 (IL-8) and mucus secretion. The cell line NCI-H292 was utilized for gene silencing of the β2-adrenergic receptor via siRNA as well as receptor blocking via ICI 118,551 and butaxamine.

**Results:**

At baseline, COPD-ALIs produced greater amounts of IL-8 than control ALIs. Olodaterol reduced RSV-mediated IL-8 secretion in both COPD and control ALIs and also significantly reduced Muc5AC staining in COPD-ALIs infected with RSV. A non-significant reduction was seen in control ALIs. Gene silencing of the β2-adrenergic receptor in NCI-H292 negated the ability of olodaterol to inhibit IL-8 secretion from both RSV infection and lipopolysaccharide stimulus, as did blocking of the receptor with ICI 118,551 and butaxamine.

**Conclusions:**

Olodaterol exhibits inherent anti-inflammatory properties on the airway epithelium, in addition to its bronchodilation properties, that is mediated through the β2-adrenergic receptor and independent of ICS usage.

## Introduction

Chronic obstructive pulmonary disease (COPD) is a highly prevalent condition that is characterized by persistent airflow limitation and is a major cause of morbidity and mortality worldwide [1, 2]. In addition to parenchymal destruction, chronic inflammation can lead to airway remodeling and narrowing, which contributes to the characteristic reduction in airflow and symptoms of cough, shortness of breath and excess sputum production present in COPD [3]. The clinical course is frequently punctuated by periods of acute symptom worsening, which are called exacerbations. These exacerbations are responsible for a large number of hospitalizations each year that exact heavy economic and societal burdens on the healthcare system [4]. The most common triggers for acute exacerbations of COPD are viral respiratory tract infections, which can also initiate chronic airway inflammation by inducing innate immune responses that activate pro-inflammatory mediators [5–7]. One of the more common pathogens is respiratory syncytial virus (RSV), which directly infects the airway epithelium [1, 5, 7].

Popular symptomatic management for patients with COPD include bronchodilators such as the long-acting β2-agonists (LABAs) which target airway epithelia and smooth muscle cells [2, 8, 9]. First approved in 2013, olodaterol (Striverdi “Respimat”) is a novel inhaled ultra-LABA with over 24 h of bronchodilatory effect [2, 10–12]. It is a potent and selective β2-adrenergic receptor (β2AR) agonist with a rapid onset of action [8, 9, 13]. By forming a stable complex with the β2ARs located in airway smooth muscles cells, olodaterol not only relaxes airway smooth muscles by stimulating adenylyl cyclase to upregulate cyclic AMP production, but also maintains broncho-protection for 24 h and therefore enables its once-daily dosing regimen [8, 14, 15]. Usage of LABAs (such as salmeterol) has been shown to decrease the risk of exacerbations in COPD patients by up to 20% compared to a placebo [16], but this is not well understood. It has been suggested that this may be due to synergy with inhaled corticosteroids, which result in an amplified anti-inflammatory effect [17], but a meta-analysis showed that LABAs were able to inhibit exacerbations in the absence of ICS as well [18]. The airway epithelium plays a key role in initiaing immune responses [19]. Therefore, we hypothesize that LABAs have their own intrinsic anti-inflammatory effects when applied to the airways of COPD patients via the airway epithelium.

## Materials and methods

### Reagents and supplies

Olodaterol hydrochloride [BI 1744 Cl] was provided by Boehringer Ingelheim Pharma GmbH & Co. KG, Germany. Interleukin (IL-8) ELISA kit [Cat# CHC1303] was purchased from Life Technologies (Carlsbad, CA, USA). Anti-Muc5AC antibody [Cat# ab24071] for immunohistochemistry was purchased from Abcam (Cambridge, United Kingdom). ICI 118,551 hydrochloride [Cat# I127-5MG] was purchased from Sigma Aldrich (St. Louis, MO, US). Butaxamine hydrochloride [B1385-50MG] was also purchased from Sigma Aldrich.

### Respiratory syncytial virus (RSV) preparation

Human Long strain, type A RSV (American Type Culture Collection (ATCC), Rockville, MD, USA) was propagated on Hep-2 (ATCC) cell monolayers as previously described [20]. The culture medium consisted of Dulbecco’s Minimum Eagle Medium (DMEM) containing heat inactivated 10% fetal bovine serum (FBS), 100 U/mL penicillin/streptomycin, non-essential amino acids and sodium pyruvate was incubated at 37 °C in a 5% CO_2_ incubator. After 4 days of culture, the condition media containing free virus was harvested and centrifuged at 10,000×*g* for 10 min at 4 °C to remove cellular debris. The clear supernatants were then pooled and concentrated by ultrafiltration through a 100 kDa cut-off membrane [Cat# UFC910008] from Millipore (Burlington, MA, USA) according to the manufacturer’s instructions. RSV infection was performed at multiplicity of infection 1 (MOI_1_) for 90 min as per previously published protocols [21]. Following viral infection, cultures were washed with PBS to remove unbound virus and exposed to air for 16 h.

### Cell culture

A total of 10 subjects (5 COPD and 5 controls) were grouped based on spirometry results according to recommendations by the Global Initiative for Chronic Obstructive Lung Disease (GOLD) [22] whereby subjects with normal lung function were considered controls. Of the five subjects in the COPD cohort and the five subjects in the non-COPD cohort, one was a non-smoker, two were former smokers and two were current smokers. The patient demographics information is shown in Table 1. Three out of the five COPD subjects were using pulmonary medications (LAMA/SABA/Prednisone, LABA/SABA, budesonide). Non-COPD subjects were not using any pulmonary medication. All subjects underwent bronchoscopy for a variety of different clinical indications including evaluation of lung nodules/masses and chronic cough. Prior to bronchoscopy, all subjects underwent thoracic computed tomography (CT) as per protocol. Using cytology brushes [Cat# 4206] from Primed Canada, human primary bronchial epithelial cells (BEC) were collected in small airways (< 2 mm in diameter), generally in the right upper or left upper lobes, far away from any nodules or masses as noted on chest CTs (usually in the contralateral lung). All experiments in this study were approved by the Research Ethics Board of University of British Columbia/Providence Healthcare (REB# H11-02713 and REB# H15-01778). Patient-derived bronchial epithelial cells (BECs) were cultured in Pneumacult-Ex medium [Cat# 05008] from STEMCELL Technologies (Vancouver, BC, Canada). Cells were seeded at 80,000 per well in a 24-well plate and differentiation was induced once the cells reached 100% confluency.

**Table 1:** BEC subject demographics information

	Non-COPD	COPD
Age (years ± SD)	66 ± 8.8	64.5 ± 9.9
Sex (male: female)	1:4	3:2
FEV_1_/FVC (Mean % ± SD)	76.5 ± 3.3	55.9 ± 11.4

NCI-H292 lung epithelial cells were purchased from the ATCC and grown in Roswell Park Memorial Institute medium (RPMI) supplemented with 10% heat-inactivated FBS. The NCI-H292 cells were cultured at 37 °C in a 5% CO_2_ humidified incubator. The media was changed three times per week and the cells were passaged once they reached 90–100% confluence. Cells were used experimentally between passages 75–90.

### ALI cell culture

Air–liquid interface (ALI) cultures were grown on 24-well Corning cell culture inserts [Cat# 3470] using Pneumacult-ALI media [Cat# 0051] from STEMCELL Technologies and protocol as previously detailed [23]. Cultures were maintained as ALIs for 21–28 days until pseudostratification of the epithelium was visualized via microscopy to contain various epithelial cell types. Medium replaced 3 times per week and the apical surface rinsed with warm phosphate buffered saline (PBS) weekly to study the effects of olodaterol treatment on the apical surface with and without RSV infections.

10 µM olodaterol treatment was administered on the apical side for 8 h, followed by aspiration of the olodaterol-containing media and exposing the cultures to air for 16 h. This pre-treatment procedure was repeated for 3 days for a total of 24 h drug exposure, to better mimic the in vivo patient regiment. ALI cultures were not exposed to olodaterol treatment on the apical side for 24 continuous hours as this would lead to de-differentiation of the pseudostratified epithelium.

### Immunohistochemical (IHC) analysis

ALI cultures with or without olodaterol and RSV treatments were processed as formalin-fixed-paraffin-embedded (FFPE) blocks, and cut into 4 μm sections. Sections were de-paraffinized in CitriSolv (Fisher Scientific, Toronto, ON, 22-143-975) and rehydrated. Expression level of mucin 5AC (Muc5AC) was detected via a Leica autostainer at 1:1000 primary anti-Muc5AC antibody dilution (AB24071, AbCam, Cambridge, MA, USA) and using the Bond Polymer Refine Red Detection kit on the Leica Bond Autostainer according to the manufacturer’s protocol.

Production of RSV viral particles in infected ALI cultures were also quantified. ALI sections were de-waxed, rehydrated, and subjected to heat-induced antigen retrieval in citrate buffer (pH 6.0, Invitrogen) for 22 min. All sections were blocked in universal protein block (Dako, Burlington, ON, Canada) at room temperature for 30 min before incubating with a 1:100 dilution of an RSV-specific antibody (NCL-RSV3, NovoCastra Labs), in Tris-buffered saline (TBS) overnight at 4 °C. The biotin-free MACH4 AP-Polymer Detection kit containing alkaline phosphatase (M4U536, Biocare Medical, Markham, ON) was then used to detect proteins of interest following manufacturer’s protocols with Warp Red Chromogen (WR806, Biocare Medical) as the substrate.

Negative controls were performed using a matched isotype antibody. Hematoxylin was used for counter-staining of the nuclei of all IHC. Colour segmentation via ImagePro Plus (Media Cybernetics, Silver Spring, MD, USA) was performed for quantification; the area of positivity was normalized to the total epithelial surface.

### Olodaterol and LPS treatment in NCI-H292 cells

NCI-H292 cells were incubated with complete media containing olodaterol for 8 h. Following pre-treatment with olodaterol, LPS was added directly into the media to a final concentration of 2 µg/mL and the culture was allowed to incubate overnight at 37 °C for 16 h before the cell-free supernatant was collected.

### siRNA transfections with olodaterol and RSV infection or lipopolysaccharide (LPS) treatment

Specific siRNAs targeting the β2AR mRNA transcript (Silencer Select s1121, s1122, s1223, s531994), scrambled non-targeting siRNA (Silencer Select 4390844) and GAPDH-targeting siRNA (Silencer Select 4390849) were purchased from Life Technologies. The β2AR siRNAs were combined in equal proportions to create a custom pool. All transfections were performed using lipofectamine RNAiMax (Life Technologies) according to the manufacturer’s instructions. Cells were plated in 24-well plates (CORNING, NY, USA) at 60,000 cells per well. Two days later, the cells were transfected with siRNA at 10 nM and incubated with the transfection complex for 24 h, after which the media was replaced with fresh complete media. After 48 h, media in the LABA treatment groups were replaced with complete media containing 10 µM olodaterol and allowed to incubate for 8 h. At 56 h, LPS was added to achieve a final concentration of 2 µg/mL and then incubated overnight. Alternatively for RSV infection, at 56 h media from all wells were removed and saved. Fresh complete media or RSV-containing media at MOI_1_ was added and allowed to incubate with the cells in a 37 °C humidified incubator for 90 min with hand shaking every 20 min to disperse the virus. After 90 min, cells were washed with PBS and aspirated before the original media was returned and the cultures allowed to incubate overnight. At 72 h, media and cell lysates were collected and centrifuged at 6000×*g* for 5 min to remove cellular debris. Total RNA was isolated using the RNeasy mini kit (Qiagen, Hilden, Germany).

### Blocking of β2AR with ICI 118,551 and butaxamine

Blocking of the β2AR in NCI-H292 cells was performed using the β2AR-selective antagonists ICI 118,551 and butaxamine. ICI 118,551 was utilized at a final concentration of 30 nM and butaxamine was utilized at a final concentration 100 nM. Cells were incubated in complete media containing the respective antagonist for 1 h prior to other treatments.

### Reverse cDNA synthesis and real-time quantitative polymerase chain reaction

cDNA was synthesized from total RNA isolates using the iScript cDNA Synthesis Kit (BioRad Laboratories, Hercules, CA, USA) and quantitative real-time PCR was performed using iTaq Universal Sybr Green Supermix (BioRad Laboratories). Subsequent data were normalized to the housekeeping gene hypoxanthine–guanine phosphoribosyltransferase (HPRT). Glycerol-3-phosphate dehydrogenase (GAPDH) was used to confirm the efficacy of the knockdown. Custom DNA primers targeting β2AR, GAPDH and HPRT cDNA transcripts were designed via PrimerBlast based on the sequence obtained from GeneBank (NM_000024.6) and are as follows: β2AR forward: 5′-ATGGGCACTTTCACCCTCTG-3′; β2AR reverse: 5′-GCTCCGGCAGTAGATAAG-GG-3′; GAPDH forward: 5′-AAGAAGGTGGTGAAGCA-GGC-3′; GAPDH reverse: 5′-CGTCAAAGGTGGAGGAGTGG-3′; HPRT forward: 5′-TGACACTGGCAAAACAATGCA-3′; HPRT reverse: 5′-GGTCCTTTTCACCAGCAAGCT-3′. Knockdown efficiency of the target genes were analyzed via the ddCt method.

### Enzyme-linked immunosorbent (ELISA) assay

Concentrations of IL-8 in supernatants collected from primary ALI cultures and NCI-H292 cells were measured by ELISA as per manufacturer’s instructions.

### Statistical analysis and data presentation

Results were analyzed using GraphPad Prism 5.0 software (GraphPad Software, San Diego, CA, USA). Graphical values are represented as the mean ± standard error of mean. Two-tailed unpaired Student t-tests were used to compare the means between two groups while ANOVA with Bonferroni correction was used for multiple group comparisons.

Where possible, data generated via primary ALI cultures were normalized to internal experiment controls to maintain consistency across experiments. This is a result of primary cells having inherent genetic variability due to being sourced from different subjects. Hence, data are presented as “fold change” in place of absolute values. NCI-H292-generated data was not normalized as cell lines lack the heterogeneity of primary cultures.

## Results

BECs from COPD and control subjects were cultured and differentiated into ALI cultures to evaluate the effects of olodaterol on the integrity and function of airway epithelial cells. Olodaterol exposure and RSV infections were performed from the apical surface to mimic in vivo drug delivery and viral infections. IL-8, a cytokine that facilitates neutrophil chemotaxis, was chosen as our surrogate marker of inflammation due to the important role it plays in COPD pathogenesis [24]. Similarly, Muc5AC was examined as it is also a specific marker of inflammation in COPD [25].

Initial experiments determined that the chosen concentration for our study, 10 µM olodaterol, did not elicit significant inflammatory or cytotoxic effects in our culture (Additional file 1: Figure S1, S2).

### COPD-ALI cultures release higher IL-8 at baseline

**Fig. 1:**
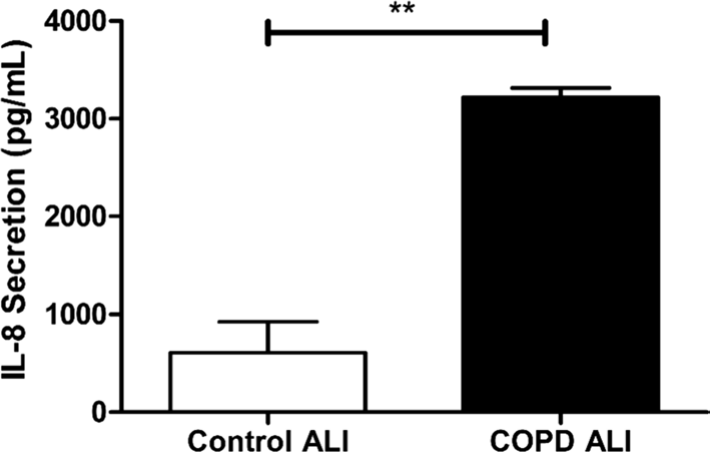
Basal concentrations of IL-8 are higher in ALI cells of COPD versus control subjects. At baseline, COPD-ALIs produced four times greater concentrations of IL-8 than control ALIs (**p < 0.01). n = 3. Data was analyzed via two-tailed unpaired Student t-test

Cell-free supernatants collected from the basal compartments of both COPD and control ALI cultures were assayed for IL-8, a chemoattractant for neutrophils that has been implicated in COPD pathogenesis. As shown in Fig. 1, constitutive IL-8 expression was four-time higher in COPD-ALI cultures versus controls (p = 0.008).

### *Mucus production in ALI cultures *via* Muc5AC staining*

**Fig. 2:**
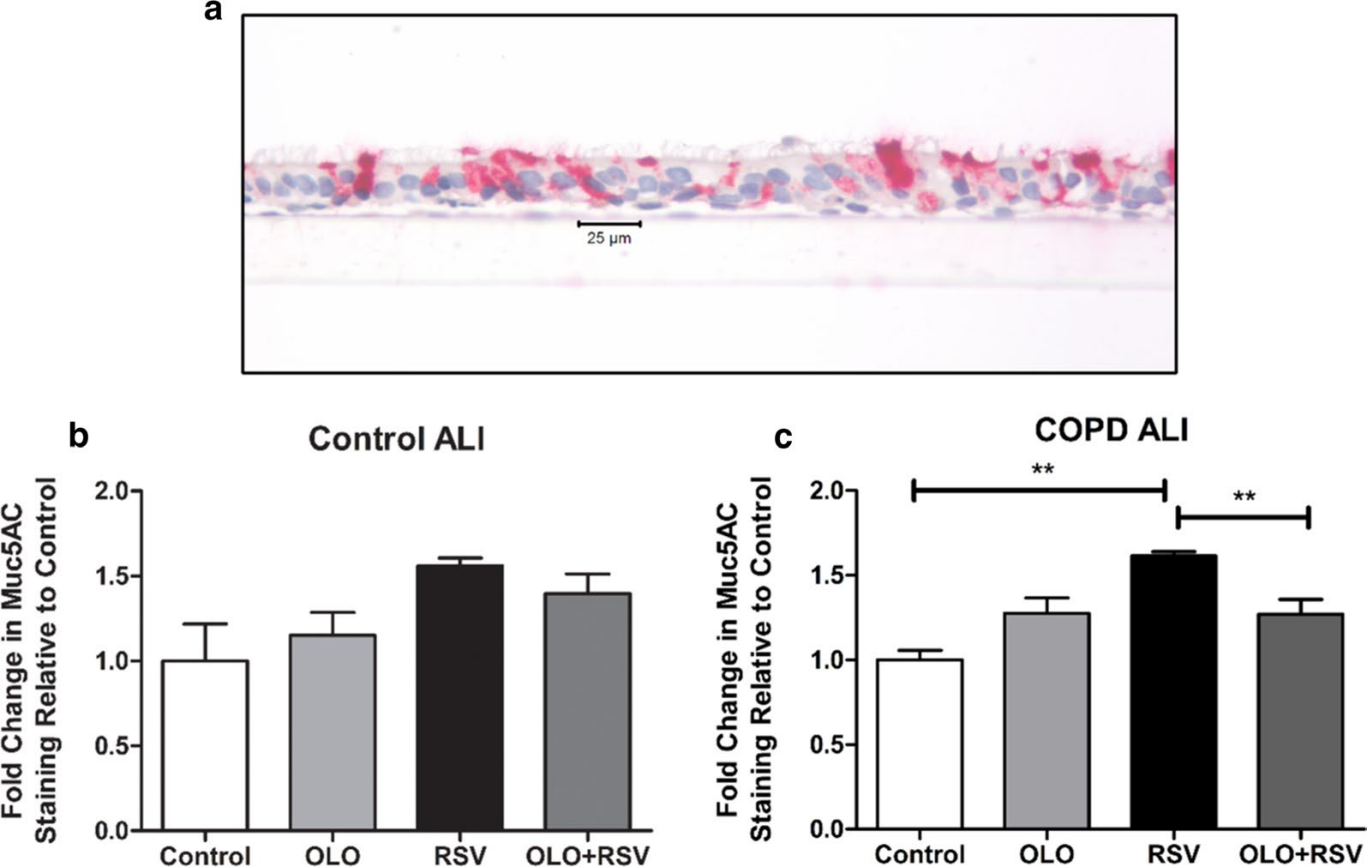
The effect of olodaterol on Muc5AC expression with and without RSV infection. Mucus production was quantified by Muc5AC staining to visualize mucins. **a** Representative image of Muc5AC staining. **b** No significant change in Muc5AC expression with or without olodaterol and RSV treatment in control-ALIs. n = 4. **c** RSV infection significantly increased Muc5AC expression compared to the control (**p < 0.01) in COPD-ALIs. Additionally, olodaterol (OLO + RSV) significantly decreased RSV-mediated Muc5AC expression (**p < 0.01). Olodaterol concentration used was 10 µM. n = 3. Data was analyzed via one-way ANOVA with Bonferroni correction

Muc5AC is a key component of airway mucus. Staining was performed on ALIs as shown in Fig. 2a. Expression of Muc5AC was higher in ALIs treated with RSV alone compared to the untreated condition in both COPD-ALIs (Fig. 2c, p < 0.01) and control ALIs (Fig. 2b, non-significant increase). In COPD-ALIs, olodaterol-treated cells demonstrated a significant reduction in RSV-mediated Muc5AC expression compared to RSV-only cells (Fig. 2c, p < 0.01). A non-significant difference was seen in non-COPD ALI cultures, likely because the COPD ALIs are already at a heightened inflammatory state and thus primed to secrete more mucins at baseline. As such, the margin of change as facilitated by olodaterol is greater and more visible in the COPD ALIs than the control ALIs.

### Olodaterol inhibited RSV-induced inflammation

**Fig. 3:**
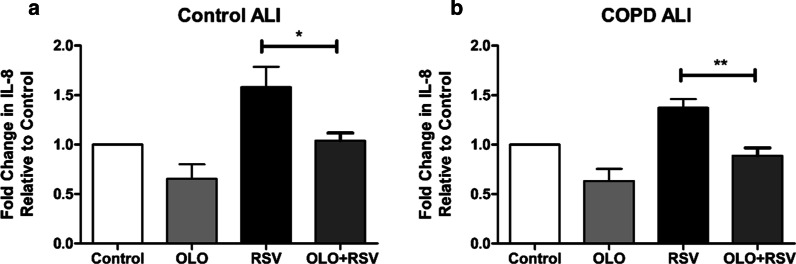
The effect of olodaterol on IL-8 secretion in ALI-cultures. **a** In control ALIs, olodaterol (OLO)-treated cultures demonstrated a significant reduction in RSV-induced IL-8 secretion compared to RSV alone (*p < 0.05). n = 4. **b** COPD-ALI cultures showed a significant decrease in RSV-mediated IL-8 secretion when pre-treated with olodaterol (OLO + RSV) compared to RSV infection alone (**p < 0.01). Olodaterol concentration used was 10 µM. n = 4. Data was analyzed via one-way ANOVA with Bonferroni correction

In both COPD and control ALI cultures, olodaterol treatment under control settings led to a slight reduction in baseline IL-8 secretion in the basal compartment compared to the untreated cultures (Fig. 3a, b), although the results were not statistically significant. This may indicate a propensity for olodaterol to inhibit baseline levels of inflammation independent of an inflammatory stimulus. Addition of olodaterol with RSV infection resulted in a significant 30% reduction in IL-8 fold change compared to RSV infection alone in the COPD-ALI (p < 0.01, Fig. 3a) and the control cultures (p < 0.05, Fig. 3b).

**Fig. 4:**
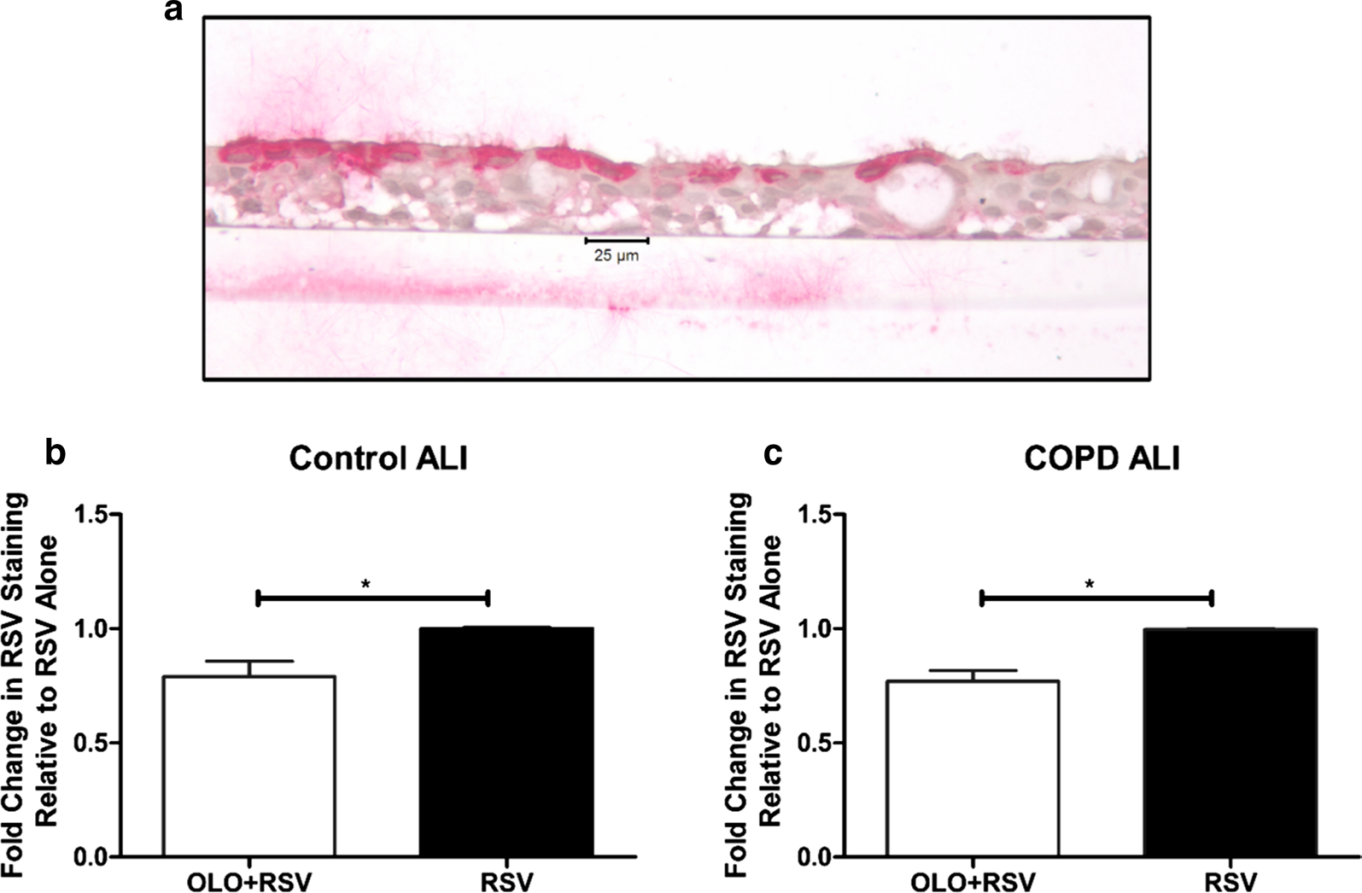
The effect of olodaterol on RSV infections. RSV staining was used to quantify RSV particles. **a** Representative image of RSV staining. **b** Control ALIs showed significantly decreased RSV staining with olodaterol treatment (OLO + RSV) compared to RSV-only infection (*p < 0.05). n = 3. **c** COPD-ALIs also showed significantly decreased RSV staining with olodaterol treatment (**p < 0.01). Olodaterol concentration used was 10 µM. n = 3. Data was analyzed via two-tailed unpaired Student t-test

Use of olodaterol exhibited a notable effect on the degree of viral infection as shown through immunohistochemical staining. A representative image of RSV staining is shown in Fig. 4a. Quantification analysis showed a significant reduction of RSV-positive cells in olodaterol-treated and RSV-infected ALIs compared to RSV alone for both COPD-ALI and control ALI cultures (p < 0.05, Fig. 4b, c). Olodaterol treatment appeared to reduce RSV infection in both COPD-ALI and control ALI.

#### β2AR is required for the inhibition of IL-8 secretion by olodaterol

NCI-H292, a lung epithelial cancerous cell line, was selected for investigation of the cellular mechanism behind olodaterol’s anti-inflammatory effect.

**Fig. 5:**
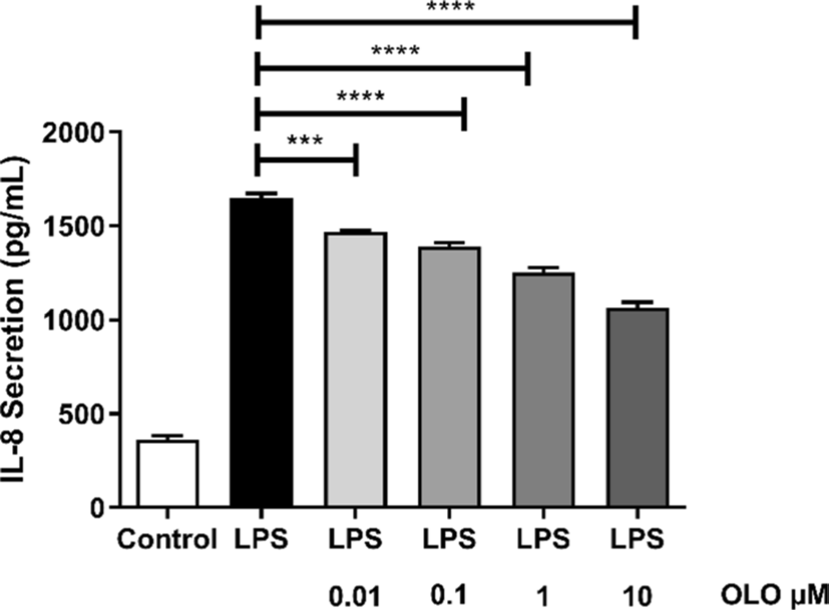
Olodaterol dose response in NCI-H292 on LPS-mediated IL-8 secretion. NCI-H292 cells were treated with 2 µg/mL of LPS and increasing log doses of olodaterol (0.01, 0.1, 1 and 10 µM). Olodaterol concentrations of 0.01 µM, 0.1 µM, 1 µM and 10 µM all significantly inhibited IL-8 secretion compared to the LPS-only treatment (***p < 0.001, ****p < 0.0001, ****p < 0.0001 and ****p < 0.0001, respectively). n = 6. Data was analyzed via one-way ANOVA with Bonferroni correction

A dose response experiment, utilizing NCI-H292 stimulated with LPS at increasing log concentrations of olodaterol, was performed and the corresponding effect on IL-8 secretion was measured. As shown in Fig. 5, there is a clear pattern of increasing IL-8 inhibition with increasing olodaterol dosage. All tested olodaterol concentrations were able to significantly inhibit LPS-mediated IL-8 secretion in a dose-dependent manner. Olodaterol at a concentration of 10 µM elicited the greatest inhibition.

**Fig. 6:**
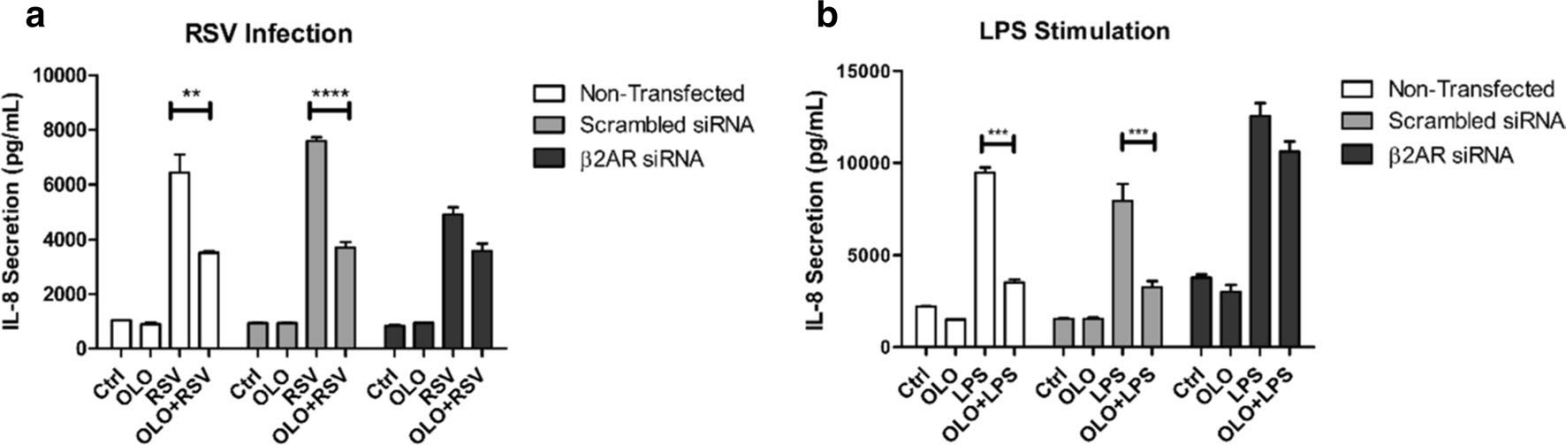
The effect of β2AR gene silencing on IL-8 secretion induced by RSV and LPS. **a** In non-transfected and scrambled siRNA-transfected cells, olodaterol inhibited RSV-mediated IL-8 secretion (OLO + RSV, **p < 0.01 and ****p < 0.0001 respectively) but not in β2AR siRNA-transfected cells. **b** Olodaterol attenuated LPS-mediated IL-8 secretion in non-transfected (OLO + LPS, ***p < 0.001) and scrambled siRNA-transfected cells (OLO + LPS, ***p < 0.001) but not in β2AR siRNA-transfected cells. n = 3. In both (**a**) and (**b**), β2AR siRNA negated olodaterol-mediated reduction of LPS-induced IL-8 secretion. Olodaterol concentration used was 10 µM. n = 3. Data was analyzed via two-way ANOVA with Bonferroni correction

To determine whether the anti-inflammatory effect observed in the ALI cultures were mediated by the β2AR, siRNAs targeting the receptor were used to elicit gene silencing to approximately 80% inhibition of the mRNA in the NCI-H292 cell line (Additional file 1: Figure S3). Both RSV and LPS was used as the inflammatory stimulus in order to investigate the effect of different stimuli on olodaterol’s inhibition of IL-8. LPS has been used previously to investigate the anti-inflammatory effect of β2-agonists in the airway cell cultures [26, 27]. To examine the role of the β2AR in the anti-inflammatory effect of olodaterol, IL-8 secretion was measured. In RSV-infected cells, the inflammatory stimuli generated high amounts of IL-8 in non-transfected and scrambled siRNA transfected conditions while treatment with 10 µM olodaterol significantly attenuated these changes (Fig. 6a, p < 0.01 and p < 0.0001, respectively). The same result was seen with LPS stimulation (Fig. 6b, p < 0.001 and p < 0.001). However, when cells were transfected with siRNA targeting β2AR, olodaterol treatment failed to significantly inhibit IL-8 secretion resulting from RSV infection and LPS stimulation, indicating that β2AR is required for the inhibition of IL-8 by olodaterol. Furthermore, the results also indicate that the effects of olodaterol are not stimuli dependent.

**Fig. 7:**
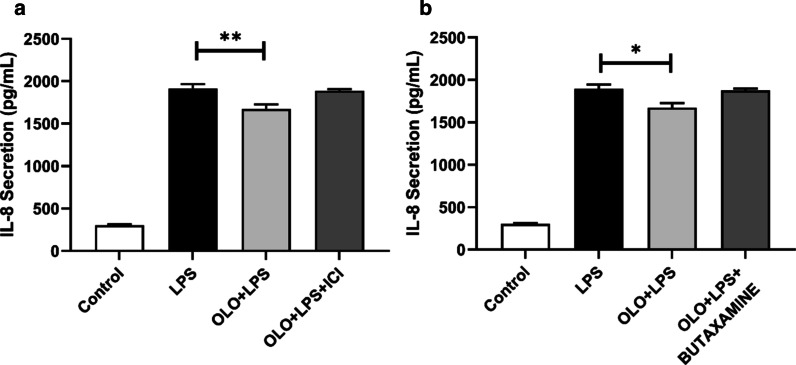
The effect of the β2AR antagonists ICI 118,551 and butaxamine on olodaterol inhibition of IL-8 secretion. NCI-H292 cells were treated with 2 µg/mL LPS with and without 0.01 µM olodaterol. To antagonize β2AR, cells were pre-treated with 30 nM of ICI 118,551 or 100 nM of butaxamine for 1 h, followed by addition of 0.01 µM olodaterol for 8 h and 2 µg/mL LPS overnight for a total of 24 h. **a** Olodaterol significantly inhibited LPS-mediated IL-8 secretion (**p < 0.01) but not in the presence of 30 nM of ICI 118,551. **b** Olodaterol also inhibited LPS-mediated IL-8 secretion (*p < 0.05) but not in the presence of 100 nM of butaxamine. n = 6. Data was analyzed via one-way ANOVA with Bonferroni correction

To further support the concept that olodaterol’s anti-inflammatory effect is mediated via β2AR, we treated NCI-H292 cells with LPS and olodaterol in the presence of ICI 118,551 and butaxamine, both of which are β2AR-selective antagonists. Although we used 10 µM in the previous experiments, this dose was “supramaximal” and as such, the compound could not be fully antagonized in receptor blocking studies. Thus, to determine the optimal dose of olodaterol required for this experiment, we conducted a dose–reponse study in which we determined that 0.01 µM olodaterol was the lowest tested concentration that was effective in inhibiting IL-8 secretion. As shown in Fig. 7a, when cells were treated with LPS and 0.01 µM olodaterol, IL-8 secretion was decreased compared to LPS-only treatment (p < 0.01). However, when cells were treated with ICI 118,551 prior to the addition of olodaterol and LPS, the effect was no longer observed. Similarly in Fig. 7b, when the experiment was repeated with butaxamine, olodaterol was unable to suppress LPS-mediated IL-8 secretion.

## Discussion

The most important and novel finding of our study was that olodaterol, an ultra-LABA, which is commonly used in COPD for bronchodilation, has significant anti-inflammatory effects in the airways of COPD patients. We showed that olodaterol was able to suppress inflammation related to RSV, a common respiratory pathogen that leads to COPD exacerbations, including suppression of IL-8 and airway mucin production/secretion and RSV viral particle production. We also showed that this anti-inflammatory effect was mediated through the β2-adrenergic receptor, the classic receptor for LABAs, as demonstrated through both siRNA gene silencing and receptor blocking experiments.

Some previously published literature have offered evidence that β2-adrenergic receptor agonists may have additional anti-inflammatory properties. This has been demonstrated previously with the SABA salbutamol, which was shown to inhibit p38 MAPK phosphorylation [28]. However, in regards to LABAs, they were first proposed to be anti-inflammatory based on the increased effectiveness of LABA/ICS combination therapy to attenuate airway inflammation compared to ICS monotherapy [29]. A popular theory was that it enhanced the anti-inflammatory effects of ICS by facilitating translocation of the glucocorticoid receptors into the nucleus [17]. However, a more recent study examining the molecular mechanisms of LABA/ICS treatment refuted this concept [30]. Several groups have noted that LABAs on their own (in the absence of glucocorticoids) demonstrate anti-inflammatory effects by inducing anti-inflammatory gene expression [30–32]. Consistent with these observations, in animal models, olodaterol has been shown to inhibit the recruitment of neutrophils to the lungs and the secretion of inflammatory mediators such as TNF-α in response to an inflammatory stimuli in the airways [27]. Furthermore, a study of 32 healthy volunteers found that salmeterol was able to reduce neutrophil recruitment and degranulation post LPS-inhalation [33]. Our findings extend these observations by demonstrating that LABAs have direct anti-inflammatory effects on COPD airway epithelial cells, which are the primary sites of disease in COPD.

We also extend previous findings by demonstrating that the anti-inflammatory effects in the airways treated with LABAs are mediated by the β2AR. While the exact downstream mechanism for interaction with inflammatory pathways is unclear, we postulate that olodaterol exerts an effect on NF-κB, a transcription factor for many cytokines including IL-8. LPS and RSV trigger an innate inflammatory response via different mechanisms but both lead to activation of NF-κB. In lung epithelial cells, RSV drives production of IL-8 through intracellular activation of Toll-like receptor 3 (via MyD88-independent pathway for later NF-κB activation) and retinoic acid-inducible gene I receptor (involved in translocation of NF-κB) [34]. LPS, meanwhile, is able to induce NF-κB mediated secretion of IL-8 through activation of TLR4 via the MyD88-dependent and MyD88-independent pathways [35]. Olodaterol may therefore play a role in stabilizing the inhibition of cytosolic NF-κB (through IκB proteins) and preventing translocation into the nucleus, leading to an inhibition of cytokine production.

Clinically, patients are prescribed olodaterol at a daily dose of 5 µg aerosolized in 22.1 µL of carrier fluid [36]. The experimental concentration of 10 µM olodaterol was chosen based on calculations of this aerosolized dose of olodaterol into the average patient’s airways’ periciliary fluid volume. The calculation for this concentration was derived from the calculations used by Dorscheid et al. to approximate the concentration of the ICS dexamethasone in the sol layer in one inhalation, assuming 10–30% deposition [37]. The resulting concentration was approximately 10 µM. To test dose-concentration, we evaluated various doses of olodaterol in the primary ALI cultures and observed that 3 µM demonstrated sub-optimal IL-8 suppression and concentrations above 10 µM were associated with notable toxicity to the cells. As the drug concentration used in this study mimics that of in vivo applications, the current study results suggest clinical relevance. It is also notable that the anti-inflammatory effect of 10 µM of olodaterol on LPS-induced IL-8 production in the airway cells is similar to that achieved by 1 µM of dexamethasone on TNF-α induced IL-8 secretion (approximately 50%) [38]. The 1 µM dose of dexamethasone approximates the dissolved concentration of one inhalation of aerosolized dexamethasone observed in the periciliary fluid of the airways [37]. In addition, it is worth noting that there is previously published work detailing the ability of the LABA formoterol in inhibiting coronavirus (HCoV-229E) infection-induced IL-8 secretion in human nasal epithelial cells [39]. Thus, there is precedence for the ability of LABAs to inhibit viral infection-induced inflammation, as was also shown in this paper. Together, the data suggest that LABAs independently exert an anti-inflammatory effect in the airways of COPD patients and may explain their salutary effects on the risk of exacerbation in COPD patients when used alone or in combination with ICS.

There were some limitations to the current study. Firstly, olodaterol appeared to also suppress the extent of RSV infection. Olodaterol may therefore have an effect in inhibiting viral replication or viral entry into the cell but the scope of this paper did not explore these potential effects. Secondly, this project used IL-8 as the biomarker of choice to monitor the anti-inflammatory effects of olodaterol. IL-8 is a known neutrophil chemoattractant and has been implicated in COPD pathogenesis. However, inflammation encompasses a wide variety of cytokines and inflammatory mediators and the effect of olodaterol on these other markers warrants further exploration. For example, it has been demonstrated that the airway epithelium constitutively secretes IL-10 [40], which exhibits a direct modulating effect on inflammation through inhibiting IL-8 gene expression [41]. Recent data from Ağaç et. al. showed that activation of the β2AR can up-regulate IL-10 [42]. Thus, olodaterol may modulate IL-8 suppression through integration with an IL-10 secreting pathway via β2AR. Thirdly, this study focused solely on the anti-inflammatory effects of olodaterol. It would also be worthwhile to explore whether this same effect of olodaterol can be extrapolated to other LABAs (eg. formoterol, salmeterol).

## Conclusion

Olodaterol demonstrates significant anti-inflammatory effects on airway epithelial cells in addition to its well-documented bronchodilation effects in the airways of COPD patients. We present evidence of olodaterol’s ability to attenuate IL-8 secretion and decrease mucus production. We also showed that the anti-inflammatory effects of olodaterol are mediated through the β2-adrenergic receptor in the airway epithelium, which is the established binding receptor of olodaterol. These observations may explain the salutary effects of LABAs in reducing exacerbations in COPD patients. More research is required to elucidate the mechanistic pathway behind these observations and whether they are applicable to other LABAs as well as their clinical translation.

## Supplementary Information

**Additional file 1: Figure S1.** Olodaterol dose response on IL-8 secretion in ALI cultures. **Figure S2. **Apical treatment of 10 µM olodaterol is non-toxic to ALI cultures. **Figure S3.** Quantification of B2AR and GAPDH mRNA transcript levels after transfection by siRNA.

## Data Availability

The datasets used and/or analysed during the current study are available from the corresponding author on reasonable request.

## Abbreviations

ALI: Air–liquid interface
β2AR: β2-Adrenergic receptor
BECs: Bronchial epithelial cells
COPD: Chronic obstructive pulmonary disease
ICS: Inhaled corticosteroid
IL-8: Interleukin-8
LABA: Long-acting beta 2 agonist
LPS: Lipopolysaccharide
MOI_1_: Multiplicity of infection 1
Muc5AC: Mucin 5AC
MyD88: Myeloid differentiation primary response 88
NF-κB: Nuclear factor kappa B
RSV: Respiratory syncytial virus
SABA: Short-acting beta agonist
TLR4: Toll-like receptor 4
