# Supplementary material for: Olodaterol exerts anti-inflammatory effects on COPD airway epithelial cells
Source: Respir Res. 2021 Feb 23;22:65. doi: 10.1186/s12931-021-01659-2 (PMC7901009; doi:10.1186/s12931-021-01659-2)
Supplement: Supplementary file 1 — Additional file 1: Figure S1. Olodaterol dose response on IL-8 secretion in ALI cultures. Figure S2. Apical treatment of 10 µM olodaterol is non-toxic to ALI cultures. Figure S3. Quantification of B2AR and GAPDH mRNA transcript levels after transfection by siRNA. [file 12931_2021_1659_MOESM1_ESM.docx]

**Supplemental:**


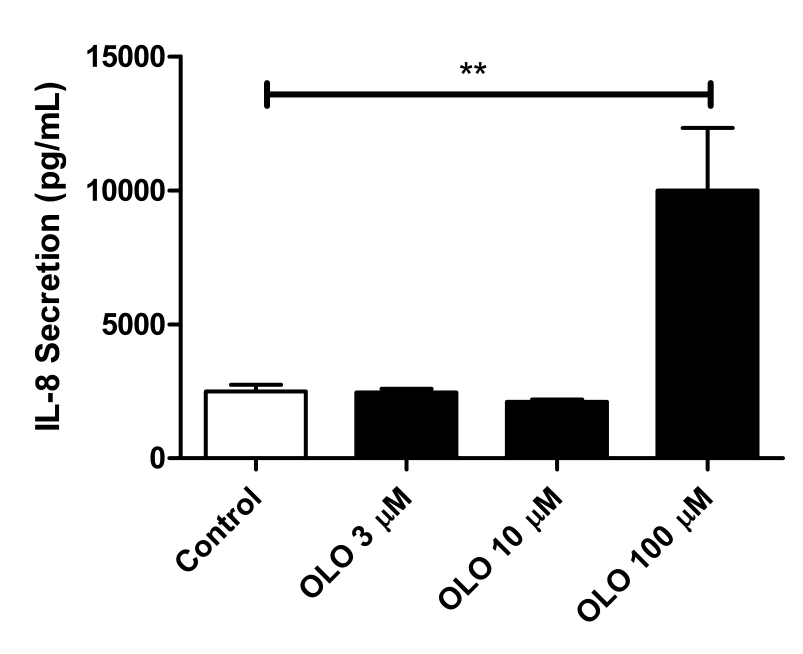


***FIGURE S1.* Olodaterol dose response on IL-8 secretion in ALI cultures.** IL-8 secretion was measured in the basal media of ALI cultures (COPD and control) treated with varying concentrations of olodaterol. Treatment with 3 µM or 10 µM olodaterol (OLO) did not increase injury response in form of IL-8 secretion compared to the non-treated control. Treatment with 100 µM of olodaterol greatly increased injury response in the form of IL-8 secretion (***p* < 0.01). n=4, mean + SEM. One-way ANOVA with Bonferroni post-test was performed.


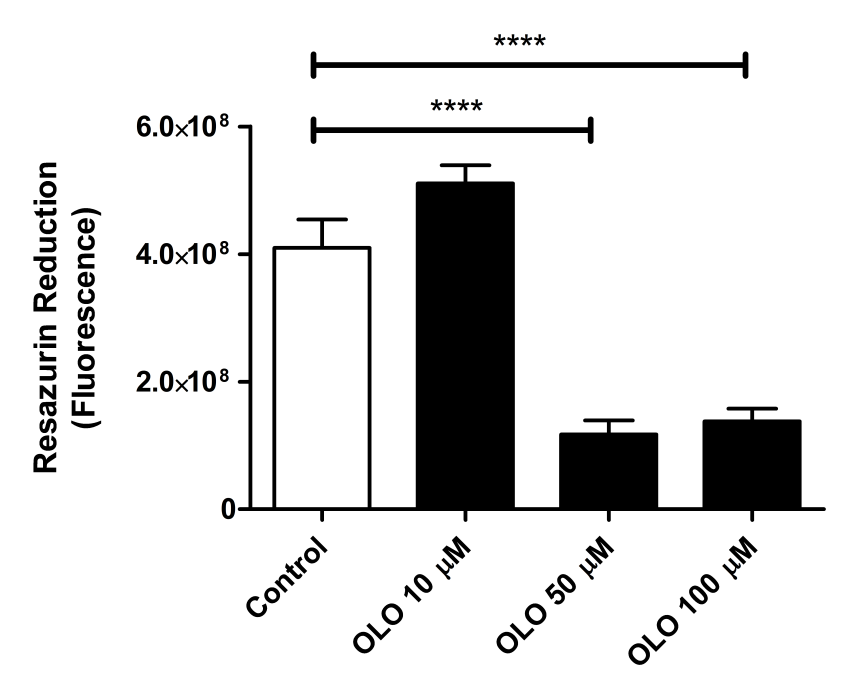


*FIGURE S2*. Apical treatment of 10 µM olodaterol is non-toxic to ALI cultures. Resazurin (alamar blue) was added to the basal compartment of olodaterol treated ALI cultures (COPD and control) at baseline. Samples of condition media were taken at 24 hour time point to quantify reduction of resazurin to resorufin (proportional to aerobic respiration). Decreased Resazurin reduction indicates decreased culture viability and increased cellular toxicity. Reduction of resazurin to resorufin was significantly decreased (*****p* < 0.0001) when ALI cultures were treated with 50 µM or 100 µM of olodaterol (OLO). n=6, mean + SEM. One-way ANOVA with Bonferroni post-test was performed.


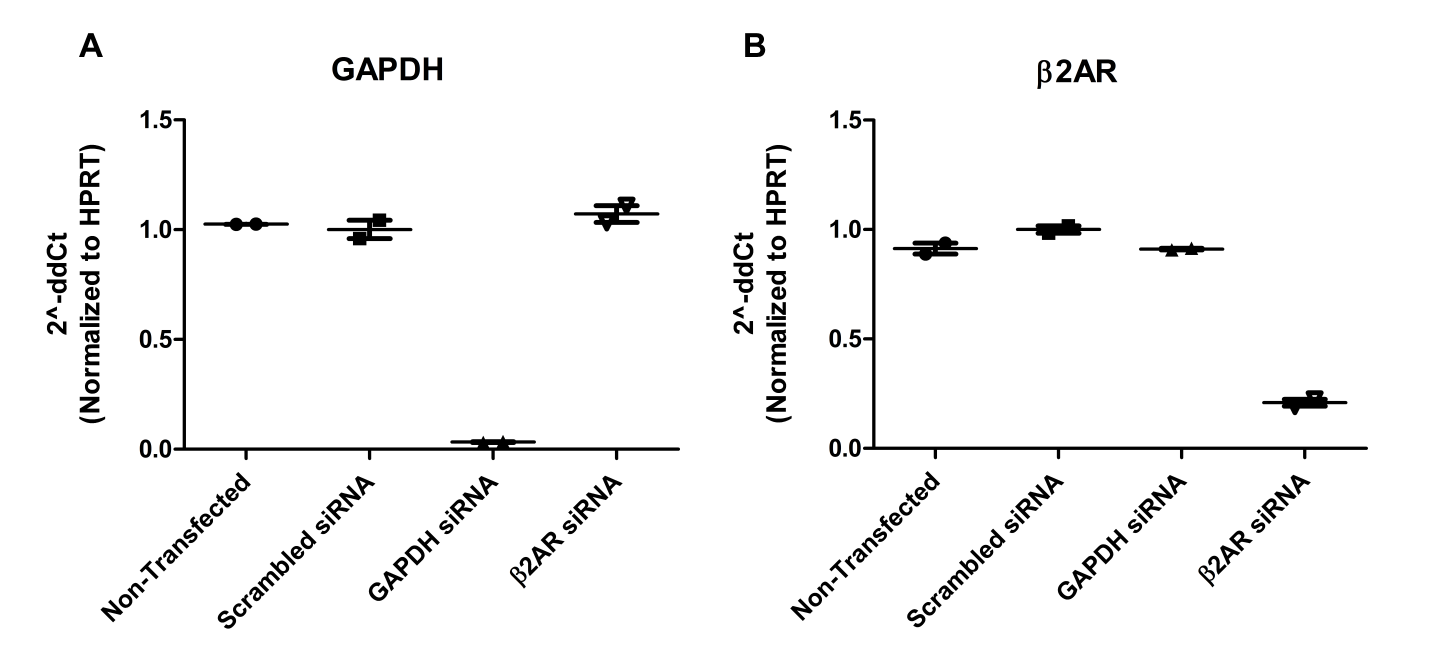


FIGURE S3. **Quantification of B2AR and GAPDH mRNA transcript levels after transfection by siRNA.** Non-targeting scrambled siRNA and siRNA targeting the B2AR or GAPDH mRNA sequences were transfected into NCI-H292 and quantified by RT-qPCR. (A) Transfection with GAPDH-targeting siRNA affected an almost 100% knockdown of GAPDH mRNA transcripts compared to non-transfected, scrambled or β2AR-targeting siRNA. (B) Transfection with B2AR-targeting siRNA facilitated an approximately 80% decrease in B2AR mRNA compared to non-transfected, scrambled or GAPDH siRNA. n=2, mean + SEM.
